# Effective Action Approach to Bose-Einstein Condensation of Ideal Gases

**DOI:** 10.6028/jres.101.049

**Published:** 1996

**Authors:** Klaus Kirsten, David J. Toms

**Affiliations:** University of Leipzig, Institute of Theoretical Physics, Augustusplatz 10, 04109 Leipzig, Germany; Department of Physics, University of Newcastle Upon Tyne, Newcastle Upon Tyne, U.K. NE1 7RU

**Keywords:** Bose-Einstein condensation, effective action, symmetry breaking, zeta functions

## Abstract

We present a short review of how the effective action formalism, well known in relativistic quantum field theory, can be used to discuss Bose-Einstein condensation of non-relativistic gases. This method lends itself very naturally to an interpretation of Bose-Einstein condensation in terms of symmetry breaking. It also allows for the definition of a very elegant regularization technique involving generalized *ζ*-functions. We show how this method can be used to recover the well known results for the free boson gas, as well as the charged boson gas in a constant magnetic field. A general criterion for interpreting Bose-Einstein condensation in terms of a phase transition with symmetry breaking is given. Finally we present an analysis of Bose-Einstein condensation in a harmonic oscillator confining potential trap, and show how the results of this simple model are in excellent agreement with experiment.

## 1. Introduction

It is now well over seventy years since the phenomenon referred to as Bose-Einstein condensation (BEC) was first predicted [1,2]. For s system of non-relativistic spin-0 bosons in three spatial dimensions, a discussion of BEC is now part of any undergraduate course in statistical mechanics. Until recently the best experimental evidence that BEC could occur in a real physical system was liquid helium, as suggested originally by London [3]. However although the behavior of liquid helium at low temperatures can be qualitatively described by the free boson gas model, the detailed behavior deviates substantially from this simple model. Physically this is of course because the effects of interactions which are neglected in the free boson gas model are important in liquid helium. More recently it was suggested [4,5] that BEC could occur for excitons in certain types of non-metallic crystals (such as CuCl for example). There is now good evidence for this in a number of experiments [6].

The most exciting experimental evidence for BEC has come from the recent observations of very cold alkali gases. BEC has now been observed to occur in gases of rubidium [7], lithium [8], and sodium [9]. These systems are very dilute and as a first approximation are well described by a boson gas model with no interactions among the atoms. The atoms are confined in a magnetic trap which can be modelled by a harmonic oscillator potential. We have recently discussed [10,11] how the occurrence of BEC is to be interpreted in such a system, and given the details of the harmonic oscillator potential trap, are able to calculate a characteristic temperature which is in excellent agreement with the values found in the experiments. This work will be reviewed briefly in Sec. 6.

The main purpose of our review is to show how the effective action formalism may be used in a straightforward and natural way to discuss BEC. The general formalism is presented in Sec. 2. In Sec. 3 we introduce a particularly useful method for defining the effective action, and show how the usual thermodynamic potential may be recovered. The interpretation of BEC as symmetry breaking is given in Sec. 4, and used to study two models: the free boson gas, and a charged boson gas in a constant, externally applied magnetic field. A general criterion for deciding if BEC will occur as a phase transition with symmetry breaking is presented in Sec. 5. In Sec. 6 BEC in a harmonic oscillator confining potential is discussed, and we describe how this model compares favourably with the experimental results on alkali gases.

## 2. General Effective Action Formalism

In this section we wish to discuss briefly the effective action approach to quantum field theory at finite temperature and density. We will then use this formalism to see how BEC may be understood in terms of symmetry breaking, since this interpretation arises in a very straightforward manner within the effective action framework. One advantage of adopting the effective action approach is that it may be applied to situations of great generality, such as curved configuration spaces of arbitrary dimension, spaces with boundaries or with complicated topologies, or situations where background gravitational or electromagnetic fields are present. Furthermore, as we will discuss in the next section, the effective action formalism allows for a very elegant mathematical regularization procedure to be used. Finally, the extension of the method from noninteracting to interacting gases may be performed in a systematic way.

Our attention will be on a system described by a nonrelativistic Schrödinger field *Ψ* with action functional

$$S[\Psi ,\Psi^{†}]=\int dt\int_{\Sigma }d\sigma_{x}\left\{\frac{i}{2}(\Psi^{†}\dot{\Psi }-{\dot{\Psi }}^{†}\Psi )-\frac{1}{2m}{\left|D\Psi \right|}^{2}-U_{1}(x){\left|\Psi \right|}^{2}\right\}.$$ (1)

(We have adopted units for which *ħ* = 1.) Here *Σ* represents the spatial configuration space. It can be any Riemannian manifold, with or without boundary; however, as our applications will be confined to flat Euclidean space ℛ*^D^*, *Σ* may be thought of as a finite box in ℛ*^D^* with periodic boundary conditions imposed on the sides of the box. The infinite box limit will be understood. d*σ_x_* represents the invariant volume element on *Σ*, which for *Σ* = ℛ*^D^* is simply given by d*σ_x_* = d*^D^x*. *D* is the dimension of the space which we keep arbitrary. *U*_1_(***x***) represents any potential, which is assumed to be time independent. ***D*** = ∇−*ie****A*** is the gauge covariant derivative, with ***A*** the vector potential describing any background electromagnetic field which might be present. We adopt the gauge choice

$$\begin{array}{cc} A_{0}=0, & \nabla \cdot A=0 \end{array}.$$ (2)

It is possible to add a self-interaction term for the Schrödinger field to Eq. (1), or generalize in other ways by considering a number of different fields. The general formalism of this subsection does not depend in any significant way on the precise form of the action Eq. (1).

In addition to the action describing the Schrödinger field, we must include the action functional for any background gravitational or electromagnetic fields which are present. In this review we will only consider the case of time independent background magnetic fields. The action for the magnetic field will be taken as

$$S_{\mathrm{em}}=\int dt\int_{\Sigma }d\sigma_{x}\left\{\frac{1}{4}F_{ij}F^{ij}-J_{\mathrm{ext}}^{i}A_{i}\right\}.$$ (3)

Here ***A****^i^* are the components of ***A***, and *F_ij_* = ∇*_i_A_j_* − ∇*_j_A_i_* is the field strength tensor. $J_{\mathrm{ext}}^{i}$ represents the components of the current $J_{\mathrm{ext}}^{i}$ responsible for setting up the background magnetic field. If we have *D* = 3, then the magnetic field vector ***B*** with components *B^i^* may be defined by *F_ij_ = ϵ_ijk_B^k^*, where *ϵ_ijk_* is the antisymmetric Levi-Civita tensor. However if we keep the spatial dimension *D* general, the magnetic field is not described by a vector and we must deal with the antisymmetric tensor *F_ij_*.

The Schrödinger field action Eq. (1) is invariant under the local gauge transformation

$$\Psi (t,x)\to e^{ie\theta (x)}\Psi (t,x),$$ (4)

A(x)→A(x)+∇θ(x), (5)

where *θ* (***x***) is an arbitrary function of the spatial coordinates ***x*** on *Σ*. Associated with any local gauge symmetry is a conserved current (defined via Noether’s theorem). In the case of Eqs. (1), (4), (5), the conserved current is

$$J=\frac{ie}{2m}(\nabla \Psi^{†}\;\Psi -\Psi^{†}\nabla \Psi )-\frac{e^{2}}{m}A{\left|\Psi \right|}^{2}.$$ (6)

The conserved charge associated with this current is

$$Q=e\int_{\Sigma }d\sigma_{x}{\left|\Psi \right|}^{2}.$$ (7)

Apart from a factor of *e*, Eqs. (6) and (7) may be seen to be the probability current, and probability of wave mechanics. Provided that we restrict the background electromagnetic field to sufficiently small values to prohibit pair production, we will have a conserved particle number

$$N=Q/e=\int_{\Sigma }d\sigma_{x}{\left|\Psi \right|}^{2}.$$ (8)

In the case of neutral systems, such as atoms confined by a magnetic trap, it is the number of particles which is conserved. We will therefore consider *N*, rather than *Q*, to be conserved in what follows.

The thermodynamics is described by the grand partition function F. In order to incorporate the conserved particle number *N* it is customary to introduce a Lagrange multiplier *μ* called the chemical potential. Then we may write

$$F=\mathrm{tr}\exp\left(-\beta (\hat{H}-\mu \hat{N})\right).$$ (9)

Here $\hat{H}$ is the Hamiltonian operator for the theory Eq. (1), and $\hat{N}$ is the number operator which is obtained from Eq. (1) by regarding *Ψ* and *Ψ*^†^ as field operators.

We will use the path integral method [12,13] to compute F. To do this it is easiest if we adopt the imaginary time formalism, where the path integral extends over all fields which are periodic in imaginary time with period *β* = 1/(*kT*). The grand partition function may be expressed as

$$F=\int [d\Psi^{†}d\Psi ]e^{-\tilde{S}},$$ (10)

where

$$\tilde{S}=S_{\mathrm{em}}+\int_{0}^{\beta }dt\int_{\Sigma }d\sigma_{x}\left\{\frac{1}{2}(\Psi^{†}\dot{\Psi }-{\dot{\Psi }}^{†}\Psi )+\frac{1}{2m}{\left|D\Psi \right|}^{2}-\mu {\left|\Psi \right|}^{2}+U_{1}(x){\left|\Psi \right|}^{2}+J_{s}^{†}\Psi +\Psi^{†}J_{s}\right\}$$ (11)

is obtained from Eq. (1) by performing the Wick rotation *t* → *–it* to imaginary time, and including the conserved particle number Eq. (8) with the Lagrange multiplier *μ*. A Schwinger [14] source *J_s_* and its complex conjugate $J_{s}^{†}$ have been introduced in order to define the effective action in a way we will describe later. These sources are also useful for obtaining the Green functions of the theory. The periodic boundary conditions imposed on the fields in the path integral ensure that the Green functions obey the usual boundary conditions for finite temperature field theory. (See Ref. [15] for example.)

It is important to keep in mind what is being held fixed when the path integral Eq. (10) is performed. The path integral is computed with the temperature *T*, volume and metric on *Σ*, chemical potential *μ*, background gauge field ***A***, and Schwinger sources *J_s_* and $J_{s}^{†}$ all regarded as fixed. By performing Legendre transformations on the appropriate variables, it is possible to obtain other functionals which hold different variables fixed. In place of the chemical potential *μ*, we wish to keep the particle number fixed. In place of the Schwinger sources *J_s_* and $J_{s}^{†}$, it proves advantageous to introduce background fields $\bar{\Psi }$ and ${\bar{\Psi }}^{†}$. Because we will only consider the transformation from $\left(\mu ,J_{s}J_{s}^{†}\right)$ to $\left(N,\bar{\Psi },{\bar{\Psi }}^{†}\right)$ we will not indicate the functional dependence on any other variables which the partition function depends on.

We will define

$$W[\mu ,J_{s}J_{s}^{†}]=-\ln F[\mu ,J_{s}J_{s}^{†}].$$ (12)

In interacting quantum field theory *W* is the generator of connected Green functions. We will eliminate the dependence on the Schwinger sources *J_s_* and $J_{s}^{†}$ by defining

$${\bar{\Psi }=\frac{\delta W}{\delta J_{s}^{†}}|}_{\mu ,J_{s}},$$ (13)

$${{\bar{\Psi }}^{†}=\frac{\delta W}{\delta J_{s}}|}_{\mu ,J_{s}^{†}},$$ (14)

and then defining the Legendre transform

$$\Gamma [\mu ,\bar{\Psi },{\bar{\Psi }}^{†}]=W[\mu ,J_{s}J_{s}^{†}]-\int_{0}^{\beta }dt\int_{\Sigma }d\sigma_{x}(J_{s}^{†}\bar{\Psi }+{\bar{\Psi }}^{†}J_{s}).$$ (15)

$\Gamma [\mu ,\bar{\Psi },{\bar{\Psi }}^{†}]$ is called the effective action. In interacting quantum field theory *Γ* is the generator of one-particle irreducible Green functions [16]. It is important to realize that when *Γ* is defined in this conventional way, it corresponds to a fixed chemical potential rather than a fixed particle number.

The thermodynamical quantities might all be expressed in terms of the effective action. We are not going to present all of them but only the particle number,

$$\begin{array}{c} N=\langle \hat{N}\rangle =\frac{1}{F}\mathrm{tr}[\hat{N}e^{-\beta (\hat{H}-\mu \hat{N})}]\\ {=-\frac{1}{\beta }\frac{\partial }{\partial \mu }W|}_{\mu ,J_{s}^{†}}, \end{array}$$ (16)

which is immediately seen to have the form

$${N=-\frac{1}{\beta }\frac{\partial \Gamma }{\partial \mu }|}_{\bar{\Psi },{\bar{\Psi }}^{†}}.$$ (17)

Furthermore, in the given formulation it can be shown [17], that in thermal equilibrium $\Gamma [\mu ,\bar{\Psi },{\bar{\Psi }}^{†}]$ should be a minimum. In particular

$$\frac{\delta \Gamma }{\delta \bar{\Psi }}{{|}_{\mu ,{\bar{\Psi }}^{†}}=0=\frac{\delta \Gamma }{\delta {\bar{\Psi }}^{†}}|}_{\mu ,\bar{\Psi }}.$$ (18)

To summarize this section, if we have an expression for the effective action *Γ*, then the effective field equations which determine the background field follow from Eq. (18). The total number of particles may be computed from Eq. (17). Although there may be easier ways of obtaining the basic quantities of physical interest in the case of free quantum field theory, the effective action formalism has a systematic expansion which can be used for interacting field theories [16]. In the next section we will discuss a practical way for obtaining the effective action.

## 3. The Generalized *ζ*-Function

The theory described by the action Eq. (11), which does not involve any self-interactions, is the simplest to deal with because the path integral Eq. (10) defining F may be done exactly, since the integrand is just a gaussian. As a result, the effective action is found to be

$$\Gamma =S_{\mathrm{em}}+\int_{0}^{\beta }dt\int_{\Sigma }d\sigma_{x}\;{\bar{\Psi }}^{†}\left[\frac{\partial }{\partial t}-\mu -\frac{1}{2m}D^{2}+U_{1}(x)\right]\bar{\Psi }+\tilde{\Gamma },$$ (19)

where

$$\tilde{\Gamma }=\ln\;\det\;\ell \left[\frac{\partial }{\partial t}-\mu -\frac{1}{2m}D^{2}+U_{1}(x)\right].$$ (20)

The first two terms in Eq. (19) may be recognized as the classical action for the background field $\bar{\Psi }$ with no Schwinger source terms. The last term of *Γ*, which we have written as $\tilde{\Gamma }$, contains the effects due to the quantum fluctuations *Ψ*′ around $\bar{\Psi }$, $\Psi =\bar{\Psi }+\Psi^{\prime }$.

We can now try to use our expression for *Γ*. The first problem we encounter is that we must obtain a more explicit result for $\tilde{\Gamma }$ by evaluating the determinant of a differential operator. The most elegant method for doing this makes use of generalized *ζ*-functions [18,19], and is motivated by analogy with the determinant of a matrix. If *M* is any Hermitian matrix, we would define det *M* to be the product of all of its eigenvalues. If *m_j_* where *j* = 1,…, *n*, are the eigenvalues, then

$$\ln\;\det\;(\ell M)=\ln\prod_{j=1}^{n}(\ell m_{j})=\sum_{j=1}^{n}\ln\;(\ell m_{j}).$$ (21)

Suppose that we define a function *ζ* (*s*) by

$$\zeta (s)=\sum_{j=1}^{n}{\left(m_{j}\right)}^{-s}.$$ (22)

By analogy with the Riemann *ζ*-function, which is $\zeta_{R}(s)=\sum_{n=1}^{\infty }n^{-s}$ for *ℜ* (*s*) > 1, the function defined in Eq. (22) is called a generalized *ζ*-function. A simple computation shows that

$$\begin{array}{cc} \zeta (0)=n, & \zeta^{\prime }(0)=-\sum_{j=1}^{n}\ln\;m_{j} \end{array}.$$ (23)

We can therefore write Eq. (21) in the form

$$\ln\;\det\;(\ell M)=-\zeta^{\prime }(0)+\zeta (0)\ln\;\ell .$$ (24)

While this does not offer any practical advantages for ordinary finite dimensional matrices, it does suggest a possible way to define ln det (*ℓM*) when *M* is a differential operator: namely, set up the eigenvalue problem for the operator, work out the eigenvalues, and define a generalized *ζ*-function as in Eq. (22). Because the eigenvalue spectrum of a differential operator such as that occurring in Eq. (20) is not bounded, in general the sum over all eigenvalues used to define *ζ* (*s*) will diverge unless we restrict *s* to some region of the complex plane. This is the same as occurs for the sum used to define the Riemann *ζ*-function $\zeta_{R}(s)=\sum_{n-1}^{\infty }n^{-s}$ which only converges for *ℜ* (*s*) > 1; however, the Riemann *ζ*-function may be defined by analytic continuation throughout the entire complex *s*-plane. (See Ref. [20] for example.) For the generalized *ζ*-function we can try to define *ζ* (0) and *ζ*′ (0) by analytic continuation from the region of the complex plane where the sum over all eigenvalues converges.

For the operator in Eq. (20) we can set up the eigenvalue problem as follows. Let *f_n_* (***x***) satisfy

$$\left[-\frac{1}{2m}D^{2}+U_{1}(x)\right]\;f_{n}(x)=E_{n}\;f_{n}(x).$$ (25)

Assume that *U*_1_(***x***) ≥ 0 so that *E_n_* ≥ 0. The eigenvalues denoted by *E_n_* in Eq. (25) may be recognized as the energy levels for the time independent Schrödinger equation. We will assume that {*f_n_* (***x***)} forms a complete set of solutions to Eq. (25) which satisfies the boundary conditions relevant to the problem, and which are normalized by

$$\int_{\Sigma }d\sigma_{x}f_{n}^{*}(x)f_{n^{\prime }}(x)=\delta_{nn^{\prime }}.$$ (26)

Because the integration over the fields *Ψ* involved only those fields which were periodic in imaginary time with period *β*, the eigenfunctions of the differential operator $\frac{\partial }{\partial t}-\mu -\frac{1}{2m}D^{2}+U_{1}(x)$ take the form *f_n_* (***x***) exp (2*πjit/β*), and the eigenvalues of this differential operator are

$$\lambda_{jn}=\frac{2\pi ij}{\beta }-\mu +E_{n},$$ (27)

where *j* = 0, ± 1, ± 2, …. The generalized *ζ*-function is defined to be

$$\zeta (s)=\sum_{j=-\infty }^{\infty }\sum_{n}{\left(\lambda_{jn}\right)}^{-s}$$ (28)

in direct analogy with Eq. (22). We will define

$$\tilde{\Gamma }=-\zeta^{\prime }(0)+\zeta (0)\ln\;\ell$$ (29)

again by analogy with Eq. (24).

Before proceeding, it is helpful to show how this definition of the effective action makes contact with the standard thermodynamic results. The usual way of studying a system involves calculating the thermodynamic potential *Ω* defined by [21]

$$\beta \Omega =\sum_{n}\ln\left[1-e^{-\beta (E_{n}-\mu )}\right].$$ (30)

Suppose that we define

$$F(s,a)=\sum_{j=-\infty }^{\infty }{\left(i\omega_{j}+a\right)}^{-s}$$ (31)

where *ω_j_* = 2*πj/β.* It was shown in Ref. [22] that

$$F(s,a)=a^{-s}+\frac{\beta^{s}}{\Gamma (s)}\sum_{n=1}^{\infty }\frac{e^{-n\beta a}}{n^{1-s}}.$$ (32)

Using this basic result, the *ζ*-function Eq. (28) may be written as

$$\zeta (s)=\sum_{n}{\left(E_{n}-\mu \right)}^{-s}+\zeta_{T}(s),$$ (33)

where

$$\zeta_{T}(s)=\frac{\beta^{s}}{\Gamma (s)}\sum_{n}\sum_{k=1}^{\infty }\frac{e^{-k\beta (E_{n}-\mu )}}{k^{1-s}}.$$ (34)

If we expand *ζ_T_* (*s*) about *s* = 0 we see that *ζ_T_* (0) = 0 and

$$\zeta_{T}^{\prime }(0)=\sum_{n}\sum_{k=1}^{\infty }\frac{e^{-k\beta (E_{n}-\mu )}}{k}=-\sum_{n}\ln\left[1-e^{-\beta (E_{n}-\mu )}\right].$$ (35)

(This is noted easily from using the expansion 1/*Γ* (*s*) = + *γs*^2^ + … valid near *s* = 0.) As *T* → 0 we see that $\zeta_{T}^{\prime }(0)\to 0$. Only the first term in Eq. (33), which has no explicit temperature dependence, will contribute to $\tilde{\Gamma }$ at *T* = 0. This contribution is associated with the zero-point energy which arises in the path integral approach [23], and disappears if we normal order the operator $\hat{H}-\mu \hat{N}$. In the ζ-function method this normal ordering is accomplished by taking *ζ* (*s*) = *ζ_T_* (*s*). Then Eq. (29) gives

$$\tilde{\Gamma }=-\zeta_{T}^{\prime }(0)=\beta \Omega ,$$ (36)

from Eqs. (30) and (35).

To summarize this section, we have shown how the effective action which governs the quantum theory may be computed for the simple Schrödinger field theory described by the classical action functional Eq. (1). The result consists of a sum of two terms; a classical part involving the background field $\bar{\Psi }$, and a quantum part given formally by Eq. (20). The formal result for $\tilde{\Gamma }$ was given meaning by the introduction of a generalized *ζ*-function, and we showed how the definition of $\tilde{\Gamma }$ in Eq. (29) was equivalent to the usual thermodynamic potential. In the next section we will show how all of this formalism may be used to discuss BEC.

## 4. BEC as Symmetry Breaking

For the free boson gas in three spatial dimensions, BEC can be understood as a phase transition. At the critical temperature characterizing the transition, the specific heat has a maximum, and the derivative of the specific heat is discontinuous. (See Refs. [24,21,25,26] for example.) In quantum field theory this phase transition can be interpreted as symmetry breaking where the symmetry which is broken is the *U*(1) gauge symmetry associated with the change in phase of the wave function. For charged particles coupled to electromagnetism, this symmetry is a local gauge symmetry; for uncharged particles the symmetry is a rigid, or global, symmetry. This was discussed in the context of relativistic field theory in cases where the background field was constant [28,27], as well as the more general case where the background field is not necessarily constant [29,30]. Nonconstant background fields are essential in cases where there is an applied magnetic field, or where there is a potential term *U*_1_(***x***).

The formalism set up in Secs. 2 and 3 is applicable whether the background field $\bar{\Psi }$ is constant or not. The equations of motion for $\bar{\Psi }$ and ${\bar{\Psi }}^{†}$ were given in Eq. (18). Using the result for the effective action obtained in Eq. (19) we find

$$\left[-\frac{1}{2m}D^{2}+U_{1}(x)-\mu \right]\bar{\Psi }=0,$$ (37)

along with the complex conjugate equation. We have used the fact that for static potentials and electromagnetic fields the background field should be independent of time: $\bar{\Psi }=\bar{\Psi }(x)$. The simplicity of this result is also a consequence of our assumption that the theory does not contain any self-interactions; this assumption results in $\tilde{\Gamma }$ containing no explicit dependence on $\bar{\Psi }$. Symmetry breaking is associated with a nonzero value for $\bar{\Psi }$. We may expand $\bar{\Psi }$ in terms of the complete set of solutions {*f_n_* (***x***)} to Eq. (25):

$$\bar{\Psi }(x)=\sum_{n}C_{n}f_{n}(x),$$ (38)

where *C_n_* are the expansion coefficients which must be determined. Substitution of Eq. (38) into Eq. (37), and using Eq. (25), results in

$$\sum_{n}C_{n}(E_{n}-\mu )f_{n}(x)=0.$$ (39)

Because the eigenfunctions obey the orthonormality condition Eq. (26), if we multiply both sides of Eq. (39) by $f_{n}^{*}(x)$ and integrate over ***x*** we have

$$C_{n}(E_{n}-\mu )=0.$$ (40)

In order that the thermodynamic potential Eq. (30) makes sense, we must have

$$\mu \leq E_{0}$$ (41)

where *E*_0_ is the lowest energy level. In terms of the generalized *ζ*-function this condition ensures that the effective action, or Helmholtz free energy, is real. It also ensures that the particle occupation numbers $1/(e^{-\beta (E_{n}-\mu )}-1)$ are all non-negative. It then follows from Eq. (40) that if *μ* < *E*_0_ ≤ *E_n_*, the only solution to Eq. (40) is for all of the expansion coefficients *C_n_* to vanish. In this case Eq. (38) becomes simply $\bar{\Psi }(x)=0$, and there is no symmetry breaking. However, if it is possible for the chemical potential *μ* to reach the critical value *μ_c_* defined by

$$\mu_{c}=E_{0},$$ (42)

then *C_n_* in Eq. (40) will be undetermined. In this case all of the *C_n_* with *n* ≠ 0 will vanish, and the background field is given by

$$\bar{\Psi }(x)=C_{0}f_{0}(x),$$ (43)

where *f*_0_(***x***) is the eigenfunction corresponding to the ground state. If *μ*_c_ = *E*_0_ is possible to attain, the symmetry is broken.

We can now make a direct link between symmetry breaking and BEC. The particle number was given in terms of the effective action by Eq. (17). If we use the result Eq. (19) for *Γ*, we may write

$$N=N_{0}+N_{1}$$ (44)

where

$$\begin{array}{c} N_{0}=-\frac{1}{\beta }\frac{\partial }{\partial \mu }\left\{S_{em}+\int_{0}^{\beta }dt\int_{\Sigma }d\sigma_{x}{\bar{\Psi }}^{†}\left[\frac{\partial }{\partial t}-\mu -\frac{1}{2m}D^{2}+U_{1}(x)\right]\bar{\Psi }\right\}\\ =\int_{\Sigma }d\sigma_{x}{\bar{\Psi }}^{†}\bar{\Psi }\\ ={\left|C_{0}\right|}^{2}, \end{array}$$ (45)

and

$$N_{1}=-\frac{1}{\beta }\frac{\partial \tilde{\Gamma }}{\partial \mu }.$$ (46)

Because *C*_0_ is associated with the ground state eigenfunction in Eq. (43), it is natural to try and associate *N*_0_ with the number of particles in the ground state, and *N*_1_ with the number of particles in excited states. If we use Eqs. (30) and (36) we have

$$N_{1}=\sum_{n}{\left[e^{-\beta (E_{n}-\mu )}-1\right]}^{-1}.$$ (47)

In the next section we will see how a general criterion can be obtained which allows us to see if it is ever possible for *μ* to reach the critical value *μ*_c_ = *E*_0_, and hence to decide if symmetry breaking can occur. For the remainder of the present section we will look at two simple applications of the results we have obtained so far.

### 4.1 Free Boson Gas

The first example we will discuss is the free boson gas in *D* spatial dimensions. The special case of *D* = 3 is treated in Refs. [24,21,25,26] using conventional statistical mechanical methods. For the case of general *D* see also Ref. [31]. We set ***A*** = 0 and *U*_1_(***x***) = 0. We take *Σ* to be a box of dimensions *L*_1_, …, *L_D_*, and will impose periodic boundary conditions on the field with the infinite box limit taken at the end. Equation (25) simplifies to

$$-\frac{1}{2m}\nabla^{2}f_{n}(x)=E_{n}f_{n}(x),$$ (48)

here, and we have

$$E_{n}=\frac{1}{2m}\sum_{j=1}^{D}{\left(\frac{2\pi n_{j}}{L_{j}}\right)}^{2}$$ (49)

where *n_j_* = 0, ±1, ±2, …. The label *n* on *E_n_* stands for the set (*n*_1_,…, *n_D_*) characterizing the energy levels. The lowest energy level is *E*_0_ = 0 here, so that the critical value found for the chemical potential is *μ*_c_ = 0. We therefore require *μ* ≤ 0. The eigenfunction *f*_0_(***x***) corresponding to *E*_0_ = 0 is

$$f_{0}(x)=V^{-1/2}$$ (50)

where *V* = *L*_1_ … *L_D_* is the volume of the box. (The factor of *V*^−1/2^ comes from the normalization condition Eq. (26)).

The generalized *ζ*-function is [see Eqs. (27) and (28)]

$$\zeta (s)=\sum_{j=-\infty n}^{\infty }\sum_{1=-\infty }^{\infty }\cdots \sum_{n_{D}=-\infty }^{\infty }{\left[i\omega_{j}-\mu +\frac{1}{2m}\sum_{i=1}^{D}{\left(\frac{2\pi n_{i}}{L_{i}}\right)}^{2}\right]}^{-s}$$ (51)

where *ω_j_* = 2*πj*/*β*. If we are interested in the infinite volume limit we may take *L*_1_,…, *L_D_* to be very large, and replace the sums over *n*_1_,…, *n_D_* with integrals. These integrals may be performed with the result

$$\zeta (s)=V{\left(\frac{m}{2\pi }\right)}^{D/2}\frac{\Gamma (s-D/2)}{\Gamma (s)}F(s-D/2,-\mu )$$ (52)

where *F*(*s*,*a*) was defined in Eqs. (31) and (32). Defining *ζ_T_* (*s*) as in Eq. (33), and making use of Eq. (36) we find (with *T* = *β*^−1^)

$$\tilde{\Gamma }=-V{\left(\frac{mT}{2\pi }\right)}^{D/2}\sum_{n=1}^{\infty }\frac{e^{n\beta \mu }}{n^{1+D/2}}.$$ (53)

From Eq. (46) we find

$$N_{1}=V{\left(\frac{mT}{2\pi }\right)}^{D/2}\sum_{n=1}^{\infty }\frac{e^{n\beta \mu }}{n^{D/2}}.$$ (54)

If *D* > 2 the sum in Eq. (54) is bounded for all *μ* ≤ 0. For large *T* we have *N*_1_ *~ T^D/^*^2^. This means that for large enough temperatures we can put any number of particles into excited states. In other words, for any value of *N*, no matter how large, we can always solve *N = N*_1_ where *N*_1_ is given by Eq. (54) for *μ* with *μ* < 0, provided that the temperature is large enough. From our discussion above this means that *C*_0_ = 0, resulting in *N*_0_ = 0 and $\bar{\Psi }=0$ so that there is no symmetry breaking.

Now consider what happens as *T* decreases. As this happens, *μ* must increase towards *μ* = 0 if we are to satisfy *N* = *N*_1_ with *N*_1_ given by Eq. (54). Eventually a critical temperature *T*_c_ is reached at which *μ* = 0. This temperature is defined by

$$N=V{\left(\frac{mT_{c}}{2\pi }\right)}^{D/2}\zeta_{R}(D/2)$$ (55)

where *ζ*_R_ is the Riemann *ζ*-function. If *ρ* = *N/V* is the density of particles, we have

$$T_{c}=\frac{2\pi }{m}{\left[\frac{\rho }{\zeta_{R}(D/2)}\right]}^{2/D}.$$ (56)

For *T* < *T*_c_ it is not possible for *μ* to decrease beyond *μ* = 0, so it remains frozen at this critical value. From Eqs. (54) and (55) we have

$$N_{1}=N{\left(\frac{T}{T_{c}}\right)}^{D/2}.$$ (57)

It is therefore not possible to accommodate all of the particles in the excited states. Because *N*_0_ = *N–N*_1_, we have

$$N_{0}=N\left[1-{\left(\frac{T}{T_{c}}\right)}^{D/2}\right]$$ (58)

as the number of particles in the ground state. Using Eq. (45) we find

$$C_{0}=N^{1/2}{\left[1-{\left(\frac{T}{T_{c}}\right)}^{D/2}\right]}^{1/2},$$ (59)

and from Eqs. (43) and (50) we have for *T < T*_c_

$$\bar{\Psi }=\rho^{1/2}{\left[1-{\left(\frac{T}{T_{c}}\right)}^{D/2}\right]}^{1/2}$$ (60)

For the special case *D* = 3, the results Eqs. (56) - (58) reproduce the standard expressions. The generalization to arbitrary *D* was given by May [32].

If *D* ≤ 2 the situation is entirely different than the one we have just described. If we try to let *μ* → 0 in Eq. (54), it is observed that the sum is not bounded. This means that we may put any number of particles into the excited states. Equivalently, for *D* ≤ 2 we can always solve *N* = *N*_1_ with *N*_1_ given in Eq. (54) for *μ* with *μ* < 0 for any temperature. It is not possible for *μ* to reach the critical value *μ* = 0 for any *T* > 0 with a finite number density of particles. This is easy to see when *D* = 2 because the sum in Eq. (54) may be easily performed to give

$$N_{1}=-V\left(\frac{mT}{2\pi }\right)\ln[1-e^{\beta \mu }].$$ (61)

Setting *N*_1_ = *N* = *ρV*, and solving Eq. (61) for *μ* gives

$$\mu =T\ln[1-e^{-\frac{2\pi \rho }{mT}}].$$ (62)

For small *T* this results in

$$\mu ≃e^{-\frac{2\pi \rho }{mT}}.$$ (63)

There is no BEC, in the same sense as BEC for the *D* = 3 gas, when *D* = 2. This agrees with the analysis of Refs. [32,33]. For *D* = 1 it is not possible to perform the sum in Eq. (54) in terms of simple functions for *μ* < 0; however, since the sum is not finite for *μ* = 0, BEC is not possible in this case.

### 4.2 BEC in a Magnetic Field

We will now consider the case where there is a constant applied magnetic field. We will allow the spatial dimension *D* to be general, but will only consider the case of a magnetic field with a single nonzero component. (The general case is more complicated and is discussed in Ref. [17].) If we choose the magnetic field to point in the *z*-direction, a suitable gauge choice for the vector potential is

A=(−By,0,…,0) (64)

where *B* is the strength of the magnetic field. Eq. (25) reads

$$\left[-\frac{1}{2m}{\left(\frac{\partial }{\partial x}+ieBy\right)}^{2}-\frac{1}{2m}\sum_{j=2}^{D}\frac{\partial^{2}}{\partial {\left(x^{j}\right)}^{2}}\right]\;f_{n}(x)=E_{n}f_{n}(x).$$ (65)

This equation is equivalent to that for a simple harmonic oscillator, and the energy levels are easily found to be [34]

$$E_{n}=(2j+1)\frac{eB}{2m}+\frac{1}{2m}\sum_{i=3}^{D}{\left(\frac{2\pi n_{i}}{L_{i}}\right)}^{2}$$ (66)

where *j* = 0, 1, 2, …; *n_i_* = 0, ±1, ±2, …; and we have again imposed periodic boundary conditions. The eigenvalues Eq. (66) are degenerate with degeneracy *eBL*_1_*L*_2_/(2*π*). The smallest energy eigenvalue is seen to be

$$E_{0}=\frac{eB}{2m}$$ (67)

and the critical value of the chemical potential is

$$\mu_{c}=\frac{eB}{2m}.$$ (68)

The generalized *ζ*-function defined in Eq. (34) is

$$\begin{array}{c} \zeta_{T}(s)=\frac{eB}{2\pi }L_{1}L_{2}\frac{T^{-s}}{\Gamma (s)}\\ \;\sum_{j=0}^{\infty }\sum_{n_{3}=-\infty }^{\infty }\cdots \sum_{n_{D}=-\infty }^{\infty }\sum_{k=1}^{\infty }\frac{e^{-k\beta (E_{n}-\mu )}}{k^{1-s}}. \end{array}$$ (69)

Taking the large box limit, and replacing the sums over *n*_3_, …, *n_D_* with integrals (which just involves a product of gaussians), and noting that the sum over *j* is just a geometric series, results in

$$\begin{array}{c} \zeta_{T}(s)=V\left(\frac{eB}{4\pi }\right){\left(\frac{mT}{2\pi }\right)}^{D/2-1}\\ \;\frac{T^{-s}}{\Gamma (s)}\sum_{n=1}^{\infty }\frac{e^{-n\beta \mu }}{n^{D/2-s}\sinh\left(\frac{neB}{2mT}\right)}. \end{array}$$ (70)

From Eq. (36) we find

$$\begin{array}{c} \tilde{\Gamma }=-\zeta_{T}^{\prime }(0)=-\;V\left(\frac{eB}{4\pi }\right){\left(\frac{mT}{2\pi }\right)}^{D/2-1}\\ \;\sum_{n=1}^{\infty }\frac{e^{-n\beta \mu }}{n^{D/2}\sinh\left(\frac{neB}{2mT}\right)}. \end{array}$$ (71)

Using this result in Eq. (46) leads to

$$\begin{array}{c} N_{1}=V\left(\frac{eB}{2\pi }\right){\left(\frac{mT}{2\pi }\right)}^{D/2-1}\\ \;\sum_{n=1}^{\infty }\frac{e^{-n\beta (\mu -\mu_{c})}}{n^{D/2-1}}{\left[1-e^{-\frac{neB}{mT}}\right]}^{-1} \end{array}$$ (72)

with *μ*_c_ given by Eq. (68).

We can now analyze whether or not BEC occurs in the same way as for the free boson gas in the previous example. First, if we use the inequality (1–e^−^*^x^*)^−1^ > 1, valid for all *x* > 0, we see that

$$N_{1}>V\left(\frac{eB}{2\pi }\right){\left(\frac{mT}{2\pi }\right)}^{D/2-1}\sum_{n=1}^{\infty }\frac{e^{-n\beta (\mu -\mu_{c})}}{n^{D/2-1}}.$$ (73)

From our earlier discussion we know that BEC is only possible if *N*_1_ remains bounded as *μ* → *μ*_c_. Because the sum in Eq. (73) is not bounded as *μ* → *μ*_c_ for *D*/2–1 ≤ 1, this inequality shows that BEC will not occur for *D* ≤ 4. This includes the physically interesting case of *D* = 3, as shown originally by Schafroth [35]. The absence of BEC for *D* ≤ 4 was given originally by May [36].

Of course one must be precise about what is meant by the absence of BEC here. What we have shown is that if BEC is interpreted to be synonymous with symmetry breaking and a phase transition in the same way as BEC occurs for the free boson gas in three dimensions, then it does not occur. On the other hand, if one interprets BEC as a sudden build-up of particles in the ground state, then it may still occur even in the absence of a phase transition. It is important to keep the definition which is chosen for BEC firmly in mind. We will return to this matter in Sec. 6. Finally, it can be shown that even though there is no phase transition, the charged Bose gas still exhibits a Meissner-Ochsenfeld effect [35]. Although we have shown that there is no phase transition if *D* ≤ 4, we have not shown that there is one for *D* > 4. To do this we must show that *N*_1_ remains bounded as *μ* → *μ*_c_. If we use the inequality

$${\left(1-e^{-\frac{neB}{mT}}\right)}^{-1}\leq {\left(1-e^{-\frac{eB}{mT}}\right)}^{-1}$$ (74)

valid for all *n* ≥ 1, then we see that

$$N_{1}<V\left(\frac{eB}{2\pi }\right){\left(\frac{mT}{2\pi }\right)}^{D/2-1}{\left(1-e^{-\frac{eB}{mT}}\right)}^{-1}\sum_{n=1}^{\infty }\frac{e^{-n\beta (\mu -\mu_{c})}}{n^{D/2-1}}.$$ (75)

For *D* ≥ 5, the sum on the RHS of this inequality remains bounded as *μ* → *μ*_c_. As in the example discussed in Sec. 4.1, this means that it is not possible to place an arbitrary number of particles in excited states if *T < T*_c_ where *T*_c_ is the solution to

$$N=\left(\frac{VeB}{mT_{c}}\right){\left(\frac{mT_{c}}{2\pi }\right)}^{D/2}\sum_{n=1}^{\infty }n^{1-D/2}{\left(1-e^{-\frac{neB}{mT_{c}}}\right)}^{-1}.$$ (76)

Unlike the case of the free boson gas, it is not possible to obtain an explicit expression for *T*_c_ in terms of the particle number, although approximate expressions can be obtained for strong and weak fields [17].

To summarize this section, we have shown the connection between BEC and symmetry breaking. The general results were illustrated with two examples. The first was the familiar case of a gas of free bosons. The second was a gas of charged bosons in a constant magnetic field. In both cases we saw how BEC interpreted as a phase transition corresponding to a breaking of the *U* (1) gauge symmetry could occur in some cases but was inhibited in others. The crucial deciding factor was the number of spatial dimensions. In the next section we will discuss a general criterion for deciding if BEC as symmetry breaking can occur.

## 5. General Criterion for BEC as Symmetry Breaking

In the last section we saw how the free boson gas did not undergo BEC, at least in the sense of a phase transition, if the spatial dimension *D* ≤ 2. If a constant magnetic field was applied to a charged gas of bosons, no BEC occurs if *D* ≤ 4. In this section we will discuss the underlying features present in these two examples which allows a unified treatment of these two cases, and in addition generalizes the analysis to a wide class of systems. We follow Refs. [37,38].

The key feature present in both examples given in the last section is that the energy levels may contain a discrete part as well as a continuous part. For the free Bose gas in *D* dimensions we had Eq. (49). In the infinite box limit *n*_1_, …, *n_D_* could be treated as continuous. Therefore *E_n_* was labelled by *D* continuous quantum numbers. BEC was found to occur for *D* ≥ 3. For the charged Bose gas in a constant magnetic field the energy levels were given by Eq. (66). This time, again in the large box limit with *n*_3_, …, *n_D_* treated as continuous, the energy levels involved (*D–*2) continuous quantum numbers, and one discrete label corresponding to the degenerate Landau levels. This time BEC occurs if *D* ≥ 5, which can be suggestively written involving the number of continuous quantum numbers as (*D–*2) ≥ 3. The feature common to both examples given in Sec. 4 is that the dimension of the space associated with the continuous labels had to be at least 3 for BEC to occur in the sense of a phase transition and symmetry breaking.

Suppose that we consider any system for which the energy levels can be expressed as the sum of a discrete part which we will denote by $E_{p}^{d}$, and a continuous part which we deal with by box normalization with the infinite box limit taken at the end as in the examples presented in the last section. We will assume that the box has dimension *q*. We may write

$$E_{n}=E_{p}^{d}+\frac{1}{2m}{\sum_{i=1}^{q}\left(\frac{2\pi n_{i}}{L_{i}}\right)}^{2},$$ (77)

where *L*_1_, …, *L_q_* are the sides of the box. Here ***p*** is just a set of labels for the discrete part of the spectrum. With the large box limit taken, the labels *n*_1_, …, *n_q_* may be regarded as continuous. The generalized *ζ*-function (34) reads

$$\begin{array}{l} \zeta_{T}(s)=\frac{\beta^{s}}{\Gamma (s)}\sum_{p}\int d^{q}n\sum_{k=1}^{\infty }\frac{e^{-k\beta (E_{n}-\mu )}}{k^{1-s}}\\ \;=\frac{V_{q}}{{\left(4\pi \right)}^{q/2}}\frac{T^{q/2-s}}{\Gamma (s)}\sum_{p}\sum_{k=1}^{\infty }\frac{e^{-k\beta (E_{p}^{d}-\mu )}}{k^{1+q/2-s}} \end{array}$$ (78)

after the integration over the continuous part of the energy spectrum has been performed.

We found that BEC with the associated phase transition only occurs if it is possible for *μ* to reach the critical value μ_c_ determined by the lowest energy level. (See the discussion around Eq. (42).) In the case of Eq. (77) we have $\mu_{c}=E_{0}=E_{0}^{d}$. Because the lowest mode is playing such a crucial role, it is advantageous to separate it off and define

$$\zeta_{T}(s)=\zeta_{T}^{\left(0\right)}(s)+\zeta_{T}^{\left(\neq 0\right)}(s)$$ (79)

where

$$\zeta_{T}^{\left(0\right)}(s)=\frac{V_{q}d_{0}}{{\left(4\pi \right)}^{q/2}}\frac{T^{q/2-s}}{\Gamma (s)}\sum_{k=1}^{\infty }\frac{e^{-k\beta (\mu_{c}-\mu )}}{k^{1+q/2-s}}$$ (80)

represents the contribution of the lowest mode to the *ζ*-function (with *d*_0_ the degeneracy), and $\zeta_{T}^{\left(\neq 0\right)}(s)$ is given by Eq. (78) but with the sum over ***p*** restricted to the nonzero modes only. We have *ζ_T_* (0) = 0 as before, and

$$\zeta_{T}^{\left(0\right)}\prime (0)=\frac{V_{q}d_{0}}{{\left(4\pi \right)}^{q/2}}T^{q/2}\sum_{k=1}^{\infty }\frac{e^{-k\beta (\mu_{c}-\mu )}}{k^{1+q/2}},$$ (81)

$$\zeta_{T}^{\left(\neq 0\right)}\prime (0)=\frac{V_{q}}{{\left(4\pi \right)}^{q/2}}T^{q/2}\sum_{p\neq 0}\sum_{k=1}^{\infty }\frac{e^{-k\beta (E_{p}^{d}-\mu )}}{k^{1+q/2}}.$$ (82)

These expressions may now be used to find the effective action $\tilde{\Gamma }$ or the thermodynamic potential *Ω* given in (36).

The advantage of separating off the lowest mode as we have done is that the argument of the exponential in Eq. (82) will remain negative even if *μ* = *μ*_c_. The consequence of this is that the sums in Eq. (82) will converge. This remains true even if we differentiate Eq. (82) with respect to *μ* to find the contribution of the excited states to the particle number given in terms of $\tilde{\Gamma }$ by Eq. (46). Therefore, whether or not a phase transition occurs representing BEC is determined by the behaviour of *ζ* ^(0)^(*s*). If we drop terms which remain finite as *μ* → *μ*_c_ we have

$$\begin{array}{c} N(\mu \to \mu_{c})≃T\frac{\partial }{\partial \mu }\zeta_{T}^{\left(0\right)}\prime (0)\\ \;≃V_{q}d_{0}{\left(\frac{T}{4\pi }\right)}^{q/2}\sum_{k=1}^{\infty }\frac{e^{-k\beta (\mu_{c}-\mu )}}{k^{q/2}} \end{array}$$ (83)

If the sum in Eq. (83) remains finite as *μ* → *μ*_c_, then the number of particles which can exist in excited states is bounded, and we get BEC and a phase transition with symmetry breaking. Clearly this can occur only for *q* > 2. If *q* ≤ 2, we can conclude that BEC does not occur, at least in the sense of a phase transition.

We can obtain much more detailed information on exactly how *N* diverges as *μ* → *μ_c_*. For *q* = 0 the sum in Eq. (83) is just a geometric series, and for *q* = 2 the sum is just the expansion of the natural logarithm. For *q* = 1 the sum may be evaluated as described by Robinson [39]. We therefore find

$$N(\mu \to \mu_{c})≃\frac{Td_{0}}{\mu_{c}-\mu }\;(\text{for}\;q=0);$$ (84)

$$N(\mu \to \mu_{c})≃\frac{1}{2}TV_{1}d_{0}{\left(\mu_{c}-\mu \right)}^{-1/2}\;(\text{for}\;q=1);$$ (85)

$$N(\mu \to \mu_{c})≃\frac{1}{2}TV_{2}d_{0}\ln\left(\frac{T}{\mu_{c}-\mu }\right)\;(\text{for}\;q=2).$$ (86)

Only that part of *N* which diverges as *μ* → *μ_c_* has been shown. The ground state when there is symmetry breaking can be determined in the way described in Sec. 4.

In this section we have shown how the occurrence of BEC is linked to the number *q* which characterizes the continuous part of the energy spectrum. For BEC to occur we require *q* ≥ 3. For the free boson gas in *D* dimensions, since *q = D*, this recovers our earlier result in Sec. 4.1. For the charged boson gas in a constant magnetic field, we have *q = D–*2, again recovering our earlier result in Sec. 4.2. The physical meaning of *q* is that it is the number of spatial dimensions for which the particles are effectively free to move. For the boson gas in a magnetic field in the *z* direction, classically the motion in the *x–y* plane is restricted to circular orbits, and it is only in the *z*-direction that the motion is free. A variety of other examples, often established by long and detailed calculations all emerge from this relatively simple analysis [38]. A similar approach may be used for relativistic gases [37,38].

## 6. BEC in Harmonic Oscillator Confining Potentials

We showed in the last section how BEC interpreted as symmetry breaking could occur only if the continuous part of the energy spectrum involved at least three continuous labels (*q* ≥ 3). A special consequence of this is that if the energy spectrum is entirely discrete, corresponding to *q* = 0, then BEC as symmetry breaking will never occur. In other words, for a system characterized by a discrete set of energy levels, if BEC does occur it cannot be interpreted as a phase transition analogously to the free boson gas in three dimensions. However, as we have discussed recently [10], it is still possible to have BEC in the sense that there is a critical temperature characterizing the system at which the number of particles in the ground state has a sudden and dramatic increase (see also Ref. [40]). This is borne out in the experiments on alkali gases at microkelvin temperatures [7–9].

The simple model we will discuss here is a system of uncharged spin-0 bosons in a harmonic oscillator confining potential. This is of phenomenological interest since it represents a model for the magnetic traps used in experiments [7–9]. We will use the action Eq. (1) with ***A*** = 0, and *U*_1_(***x***) the harmonic oscillator potential

$$U_{1}(x)=\frac{1}{2}m(\omega_{1}^{2}x^{2}+\omega_{2}^{2}y^{2}+\omega_{3}^{2}z^{2}).$$ (87)

(We will only consider 3 spatial dimensions here although the analysis may be generalized in an obvious way to any number of dimensions.) The energy levels are simply

$$E_{n}=(n_{1}+\frac{1}{2})\hbar \omega_{1}+(n_{2}+\frac{1}{2})\hbar \omega_{2}+(n_{3}+\frac{1}{2})\hbar \omega_{3},$$ (88)

where *n* = (*n*_1_, *n*_2_, *n*_3_,) with *n_i_* = 0, 1, 2, … and we have reinstated the explicit *ħ* dependence. The lowest energy level, which determines the critical value of the chemical potential as in Eq. (42), is

$$\mu_{c}=E_{0}=\frac{1}{2}\hbar (\omega_{1}+\omega_{2}+\omega_{3}).$$ (89)

The total number of particles is given by the usual Bose distribution function

$$N=\sum_{n_{1}=0}^{\infty }\sum_{n_{2}=0}^{\infty }\sum_{n_{3}=0}^{\infty }{\left\{e^{\beta (E_{n}-\mu )}-1\right\}}^{-1}.$$ (90)

Although it is possible to proceed with the anisotropic case, our results are most easily illustrated for the isotropic harmonic oscillator with *ω*_1_ = *ω*_2_ = *ω*_3_ = *ω*. (The general anisotropic case is dealt with in Ref. [11].) For the isotropic oscillator, the triple sum in Eq. (90) becomes the simpler result

$$N=\sum_{l=0}^{\infty }\frac{1}{2}(l+1)(l+2){\left\{e^{(l+\epsilon )x}-1\right\}}^{-1}$$ (91)

where we have defined the dimensionless variables *x* and *ϵ* by

$$x=\frac{\hbar \omega }{kT},$$ (92)

$$\mu =\hbar \omega (\frac{3}{2}-\epsilon ).$$ (93)

Since $\mu_{c}=\frac{3}{2}\hbar \omega$, a phase transition with the associated symmetry breaking occurs only if it is possible for *μ* to reach the value *ϵ* = 0. Our general analysis of the last section shows that this can never happen; however, for this particular example we can show this another way. If we expand

$${\left\{e^{(l+\epsilon )x}-1\right\}}^{-1}=\sum_{n=1}^{\infty }e^{-n(l+\epsilon )x},$$ (94)

then the sum over *l* in Eq. (91) is easily done with the result

$$N=\sum_{n=1}^{\infty }e^{-n\epsilon x}{\left(1-e^{-nx}\right)}^{-3}.$$ (95)

For *x* > 0 we have (1–e^−^*^nx^*)^−3^ > 1, so that

$$N>\sum_{n=1}^{\infty }e^{-n\epsilon x}={\left(e^{\epsilon x}-1\right)}^{-1}.$$ (96)

As we let *ϵ* → 0, which should be the signature for BEC, it is seen that *N* increases without bound. This is quite different from the free boson gas in three dimensions where the number of particles is bounded. This means that regardless of the temperature, it is always possible to solve Eq. (95), or equivalently Eq. (91), for *ϵ* given any finite *N*, with *ϵ* > 0. It is never possible for this system to attain the limit *ϵ* = 0 for any *T* > 0 and any finite number of particles. The fact that a Bose gas in a harmonic oscillator potential does not condense in the same way as a free boson gas was noted originally in Ref. [41].

This points out a fundamental difference between our analysis and other treatments [42,40,43]. We have treated the energy spectrum for the harmonic oscillator as discrete with the ensuing sums. The physically interesting case occurs when *ω*/2*π*, 100 Hz, and *T* ~ *μ* K. In this case, *x* as defined in Eq. (92) is small. A plausible approach is to argue that for *x* << 1, it is justified to replace a sum such as Eq. (95) with an integral. This is tantamount to regarding the energy levels as continuous rather than discrete, and the analysis of Sec. 5 shows that the underlying physics is crucially dependent on the number *q* of continuous dimensions in the energy spectrum. Any approximation which effectively changes *q* from 0 to 3 is therefore suspect.

If the correct behaviour for small *x* is desired, the only safe approach is to deal with the exact sum Eq. (95). The result in Eq. (95) does not converge very rapidly for small *x*, nor does it display in any transparent way the behaviour at small *x*. However, it is possible to convert Eq. (95) into a contour integral (which is an exact result, not an approximation), and by deforming the contour of integration in an appropriate way obtain at least an asymptotic expansion for some appropriate range of the parameters. The details of this procedure are described in Ref. [11]. We will illustrate the general technique for the number of particles as given in Eqs. (91) or (95).

The aim is to obtain an asymptotic expansion of *N* valid for small *x* and small *ϵ*. We can do this by making use of the Mellin-Barnes representation for the exponential:

$$e^{-\nu }=\frac{1}{2\pi i}\int_{c-i\infty }^{c+i\infty }d\alpha \Gamma (\alpha )\nu^{-\alpha }$$ (97)

valid for *ℜ* (*ν*) > 0 and *c* ∈ ℛ with *c* > 0. Equation (97) is easily proven by closing the contour in the left hand of the complex plane, enclosing the simple poles of *Γ* (*α*) at *α* = −*n*, *n* = 0, 1, 2, … with residue $\frac{{\left(-1\right)}^{n}}{n!}$. The residue theorem immediately gives the Maclaurin series for e^−ν^. From Eqs. (91) and (94) we have

$$\begin{array}{c} N=\sum_{n=1}^{\infty }\sum_{l=0}^{\infty }\frac{1}{2}(l+1)(l+2)e^{-n(l+\epsilon )x}\\ =\frac{1}{2\pi i}\sum_{n=1}^{\infty }\sum_{l=0}^{\infty }\frac{1}{2}(l+1)(l+2)\\ \times \int_{c-i\infty }^{c+i\infty }d\alpha \Gamma (\alpha )n^{-\alpha }x^{-\alpha }{\left(l+\epsilon \right)}^{-\alpha }. \end{array}$$ (98)

The order of summation and integration may be interchanged provided that we deform the contour of integration first so that *c* > 3. The sum over *n* can now be done in terms of the Riemann *ζ*-function, and the sum over *l* in terms of the generalized, or Hurwitz, *ζ*-function *ζ_H_* (*s*, *a*) defined by Ref. [20]

$$\zeta_{H}(s,a)=\sum_{n=0}^{\infty }{\left(n+a\right)}^{-s}$$ (99)

for ℜ (*s*) > 1 and 0 < *a* ≤ 1. (The Riemann *ζ*-function is the special case *ζ_R_* (*s*) = *ζ_H_* (*s*, 1).) The sums in the original expression for the particle number have now been performed exactly with no approximations, and we have obtained an integral representation for the particle number:

$$\begin{array}{l} N=\frac{1}{2\pi i}\int_{c-i\infty }^{c+i\infty }d\alpha \Gamma (\alpha )x^{-\alpha }\zeta_{R}(\alpha )\frac{1}{2}\{\zeta_{H}(\alpha -2,\epsilon )\\ +(3-2\epsilon )\zeta_{H}(\alpha -1,\epsilon )+(1-\epsilon )(2-\epsilon )\zeta_{H}(\alpha ,\epsilon )\}. \end{array}$$ (100)

Closing the contour in the left half of the complex plane and use of the residure theorem now leads to an asymptotic expansion of *N* in powers of *x*:

$$N≃\frac{\zeta_{R}(3)}{x^{3}}+\left(\frac{3}{2}-\epsilon \right)\frac{\zeta_{R}(2)}{x^{2}}+\frac{1}{\epsilon x}+O\left(\frac{\ln x}{x}\right).$$ (101)

It is possible to extend the range of validity of the expansion to larger values of *ϵ*. This can be done by treating the first (*l* = 0) term in Eq. (91) separately and applying the contour integral procedure we have just described to the remaining terms. The result is

$$N≃\frac{1}{e^{\epsilon x}-1}+\frac{\zeta_{R}(3)}{x^{3}}+\left(\frac{3}{2}-\epsilon \right)\frac{\zeta_{R}(2)}{x^{2}}+O\left(\frac{\ln x}{x}\right).$$ (102)

which is more accurate than Eq. (101) for larger *ϵ*. Similar expansions may be obtained for other thermodynamic functions [10,11]. For the internal energy we find

$$\frac{U}{\hbar \omega }=\frac{3\zeta_{R}(4)}{x^{4}}+\left(\frac{9}{2}-3\epsilon \right)\frac{\zeta_{R}(3)}{x^{3}}+\frac{13\zeta_{R}(2)}{4x^{2}}+\frac{3}{2\epsilon x}+O\left(\frac{\epsilon }{x^{2}},\frac{1}{x}\right).$$ (103)

For the specific heat we find

$$\begin{array}{c} C/k=\frac{12\zeta_{R}(4)}{x^{3}}+\frac{9\zeta_{R}(3)}{x^{2}}+\frac{2\zeta_{R}(2)}{x}-\frac{12\epsilon \zeta_{R}(3)}{x^{2}}\\ -\frac{9\epsilon^{2}\zeta_{R}{\left(3\right)}^{2}}{x^{4}}-\frac{18\epsilon^{2}\zeta_{R}(2)\zeta_{R}(3)}{x^{3}}\\ +\frac{9\epsilon^{4}\zeta_{R}(2)\zeta_{R}{\left(3\right)}^{2}}{x^{6}}+O\left(\ln\;x,\frac{\epsilon }{x}\right). \end{array}$$ (104)

These analytic results can be shown to be in excellent agreement with numerical results obtained from an evaluation of the exact sums in the range in which they are valid (small *x* and small *ϵ*).

In a similar way we are able to treat the anisotropic oscillator potential. Introducing the geometric mean *Ω* = (*ω*_1_ *ω*_2_ *ω*_3_)^1/3^ of the oscillator frequencies, we found for the particle number

$$N≃\frac{1}{e^{\left(\hbar \beta \epsilon /3)(\omega_{1}+\omega_{2}+\omega_{3}\right)}-1}+{\left(\frac{kT}{\hbar \Omega }\right)}^{3}\zeta_{R}(3)+\gamma {\left(\frac{kT}{\hbar \Omega }\right)}^{2}\zeta_{R}(2),$$ (105)

with

$$\gamma =\frac{1}{2}{\left(\omega_{1}\omega_{2}\omega_{3}\right)}^{2/3}\left(\frac{1}{\omega_{1}\omega_{2}}+\frac{1}{\omega_{1}\omega_{3}}+\frac{1}{\omega_{2}\omega_{3}}\right).$$ (106)

Analogous results for *U* and *C* are given in [11].

**Fig. 1:**
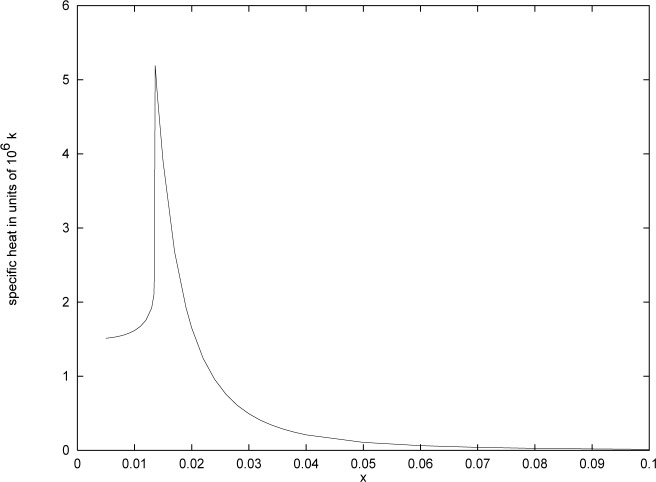
The specific heat in units of *k* as a function of *x* = *ħω*/(*kT*). The total number of particles is *N* = 5×10^5^ and *ω*/2*π* = 416 Hz.

Because there is no sharp phase transition, identifying a critical temperature is problematical. One approach which has been used in finite volume systems [44,45] is to calculate the maximum of the specific heat, and identify the temperature at which the maximum occurs with the BEC temperature. It is very difficult to obtain reliable analytic expressions for the specific heat in the region of the maximum for this model. However it is possible to compute the specific heat numerically, and the result is shown in Fig. 1.

We have chosen *ω*/2*π* = 416 Hz to be the geometric mean of the frequencies in the sodium experiment [9]. The number of particles *N* = 5×10^5^ is also taken from the sodium experiment. The maximum in the specific heat occurs for *x* ≃ 0.0136, corresponding to a temperature of *T* ≃ 1.47 *μ*K. This is in remarkably good agreement with the temperature of 2 *μ*K quoted in Ref. [9].

**Fig. 2:**
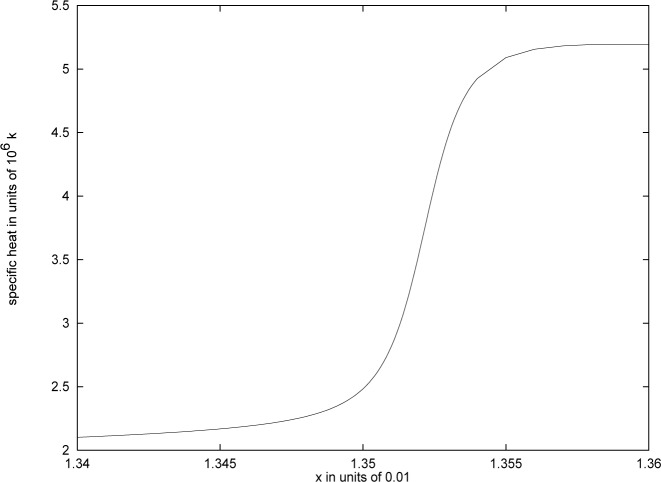
The specific heat in units of *k* as a function of 100*x* where *x* = *ħω*/(*kT*).

Another difference between our results and earlier work [42,43] is that we find the specific heat to be smooth and continuous at its maximum. A closeup of the specific heat in a neighbourhood of its maximum is shown in Fig. 2. The discontinuous behaviour found in Refs. [42,43] is due to approximating sums with integrals, which as stated earlier is not a reliable approximation.

That the specific heat is continuous has also been realized by the authors of Ref. [40]. The question of whether or not one can actually distinguish in an experiment a drop as seen in Fig. 2 from a genuine discontinuity has also been addressed there. They improved the analysis of [42,43] by taking into account a suitable density of states. In their procedure a parameter depending on the oscillator frequencies had to be determined numerically. Our above described procedure gives the complete analytical dependence, see Eq. (106).

Another possibility for obtaining an estimate of the critical temperature for BEC consists of examining the population of the ground state. The first term on the RHS of Eq. (102) may be observed as the number of particles in the ground state. (Put *k* = 0 in Eq. (91).) The remaining terms then give the number of particles in excited states. We could define the critical temperature to be the temperature at which a specified fraction of the total number of particles are in the ground state. If we define

$$N_{0}=fN,$$ (107)

where

$$N_{0}={\left(e^{\epsilon x}-1\right)}^{-1},$$ (108)

this fixes *ϵ*, and hence the chemical potential, in terms of *x* and *N* by

$$\epsilon x=\ln\left(1+\frac{1}{fN}\right).$$ (109)

**Fig. 3:**
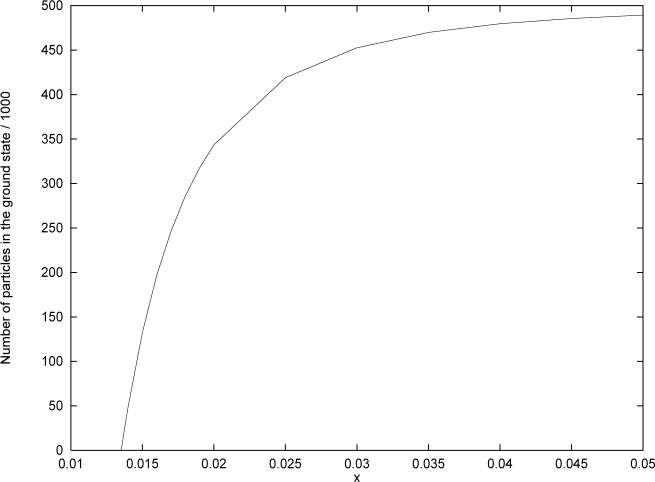
The number of particles in the ground state as a function of *x.*

For a large number of particles, *ϵ* would be expected to be small. If we use Eq. (102) we also have

$$(1-f)N≃\zeta_{R}(3)x^{-3}+\frac{3}{2}\zeta_{R}(2)x^{-2}$$ (110)

which gives a cubic equation for *x*. This may be solved in a straightforward way. (A more accurate result may be obtained by including more terms in the asymptotic expansion [Eq. (102)] beyond those indicated [11]). With *N* = 5×10^5^ we find *x* ≃ 0.0136 for *f* = 1/100; *x* ≃ 0.014 for *f* = 1/10; *x* ≃ 0.017 for *f* = 0.5; and *x* ≃ 0.0373 for *f* = 0.95. For the purpose of comparison, we have computed the ground state population numerically. The result is shown in Fig. 3. The approximate result for the number is in very good agreement with the exact result.

We have worked out the maximum in the specific heat for the two cases *N* = 2×10^5^ and *N* = 2×10^4^, and find that it corresponds to *x* ≃ 0.0185 and *x* ≃ 0.0408, respectively. Using the oscillator frequencies given in Ref. [8] we find the temperature at which the specific heat has a maximum is *T* ≃ 380 nK, in good agreement with the range of 100 nK to 400 nK for the experiment. For the case of rubidium with *N* = 2×10^4^ we find *T* ≃ 71 nK if we use the oscillator frequencies of Ref. [7]. If we use the frequencies given in Ref. [46] for the strong trap we find *T* ≃ 124 nK, again in close agreement with the experiment. We can also compare the results for the maximum in the specific heat to the fraction in the ground state found from Eq. (110). For the case *N* = 2×10^5^, we find *x* ≃ 0.0185 for *f* = 0.01; *x* ≃ 0.0191 for *f* = 0.1; *x* ≃ 0.0233 for *f* = 0.5; and *x* ≃ 0.051 for *f* = 0.95. If we take *N* = 2×10^4^ we find *x* ≃ 0.0404 for *f* = 0.01; *x* ≃ 0.0417 for *f* = 0.1; *x* ≃ 0.051 for *f* = 0.5; and *x* ≃ 0.114 for *f* = 0.95. In all three cases, the maximum in the specific heat occurs when only about 1 % of the particles are in the ground state. The specific heat maximum is therefore a good indicator of the onset of BEC.

For the case that BEC in the sense of a phase transition occurs, the critical temperature is the temperature at which the ground state starts to become populated. (See Eq. (58) for the free boson gas.) Because we have seen that the specific heat maximum also corresponds to the point at which the ground state population begins to grow, we believe that this gives a good and reliable indicator for the onset of BEC in neutral atoms trapped by a confining potential.
